# Supplementary material for: Understanding the Function and Mechanism of Zebrafish Tmem39b in Regulating Cold Resistance
Source: Int J Mol Sci. 2022 Sep 28;23(19):11442. doi: 10.3390/ijms231911442 (PMC9569763; doi:10.3390/ijms231911442)
Supplement: Supplementary file 1 [file ijms-23-11442-s001.zip › Supplementary Figures.pdf]

Supplementary Figures

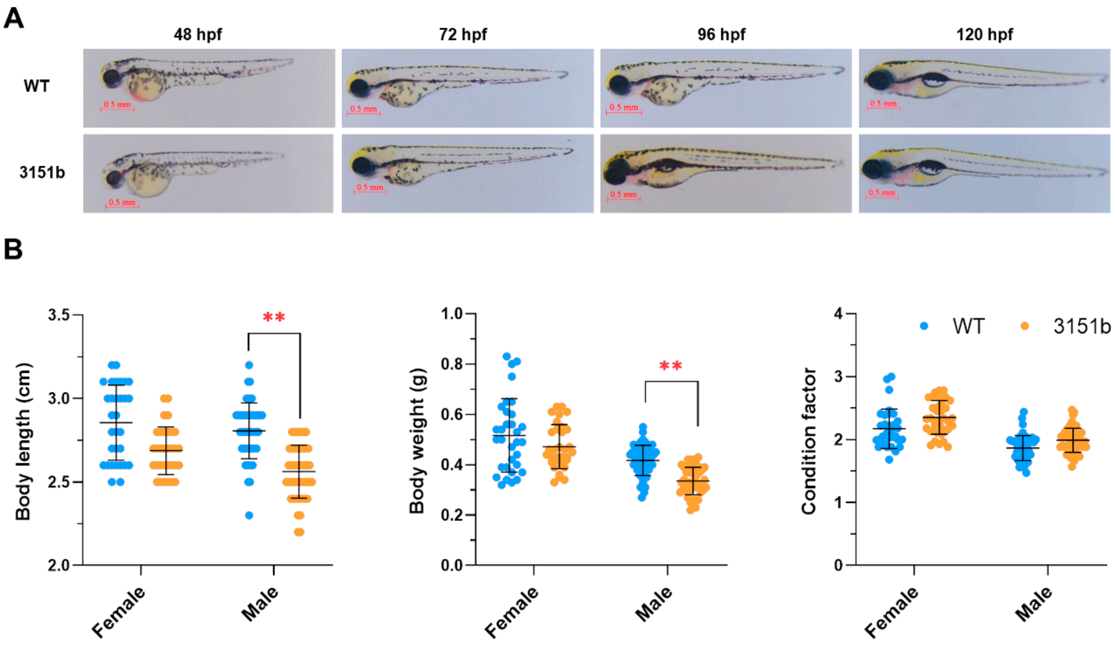

Figure S1. Effects of *tmem39b*-knockout on development and growth of zebrafish. (A) *tmem39b* mutation has no effects on early development of zebrafish. (B) Knockout of the *tmem39b* gene significantly decreased body size of the adult males. **, $p \leqslant 0.01$.

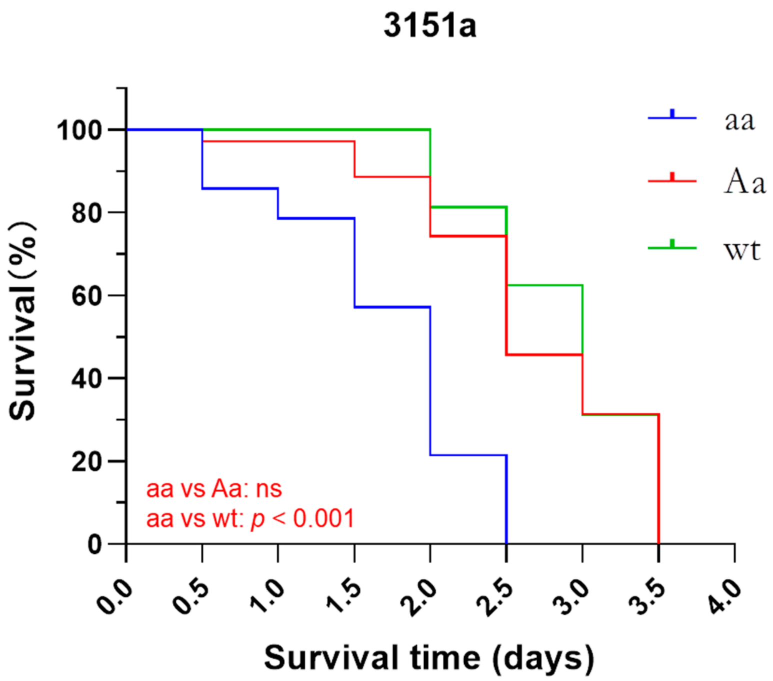

Figure S2. Comparison of cold resistance between the zko3151a mutants and the WT animals. Survival time of the adult fish for different genotypes (WT, heterozygous and homozygous) under 10 ºC lethal cold exposure.

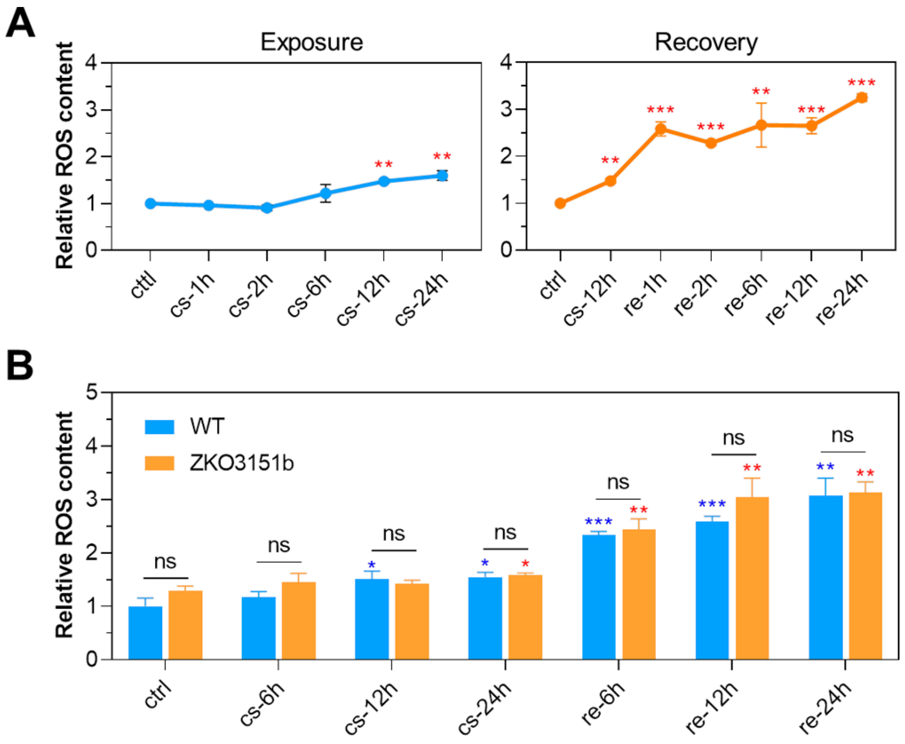

Figure S3. Mutation of *tmem39b* has no effects on the level of reactive oxygen species (ROS). (A) ROS level of the WT zebrafish larvae during cold exposure and the recovery phase after exposure. (B) *tmem39b* mutants demonstrate no difference in ROS level in comparison with the WT fish during both the exposure and recovery processes. The data are shown as mean ± SD. Means of different treatments were compared with the corresponding ctrl values. The difference between WT and zko3151b mutants at each condition was also analyzed. *, $p \leqslant 0.05$; **, $p \leqslant 0.01$; ***, $p \leqslant 0.001$; ns, not significant (n = 4).
